# *N*-[(*E*)-4-Chloro­benzyl­idene]-2,4-dimethyl­aniline

**DOI:** 10.1107/S1600536811026109

**Published:** 2011-07-06

**Authors:** Hoong-Kun Fun, Ching Kheng Quah, S. Viveka, D. J. Madhukumar, D. Jagadeesh Prasad

**Affiliations:** a X-ray Crystallography Unit, School of Physics, Universiti Sains Malaysia, 11800 USM, Penang, Malaysia; b Department of Chemistry, Mangalore University, Karnataka, India

## Abstract

The title mol­ecule, C_15_H_14_ClN, exists in a *trans* configuration with respect to the C=N bond [1.2813 (16) Å]. The dihedral angle between the benzene rings is 52.91 (6)°. The crystal structure is stabilized by weak inter­molecular C—H⋯π inter­actions.

## Related literature

For general background to and the pharmacological activity of Schiff base compounds, see: Ittel *et al.* (2000 ▶); Shah *et al.* (1992 ▶); Cimerman *et al.* (2000 ▶); Pandeya *et al.* (1999 ▶); More *et al.* (2001 ▶); Cimerman & Stefanac (2001 ▶); Galic *et al.* (1997 ▶). For the stability of the temperature controller used in the data collection, see: Cosier & Glazer (1986 ▶). For standard bond-length data, see: Allen *et al.* (1987 ▶).
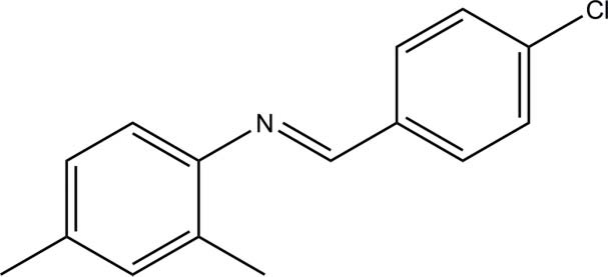


## Experimental

### 

#### Crystal data

C_15_H_14_ClN
*M* *_r_* = 243.72
Monoclinic, 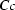

*a* = 7.2852 (1) Å
*b* = 15.2715 (2) Å
*c* = 11.5382 (1) Å
β = 96.304 (1)°
*V* = 1275.93 (3) Å^3^
*Z* = 4
Mo *K*α radiation
μ = 0.28 mm^−1^
*T* = 100 K
0.40 × 0.24 × 0.20 mm

#### Data collection

Bruker SMART APEXII CCD area-detector diffractometer
Absorption correction: multi-scan (*SADABS*; Bruker, 2009 ▶) *T*_min_ = 0.898, *T*_max_ = 0.946
14547 measured reflections
3975 independent reflections
3800 reflections with *I* > 2σ(*I*)
*R*_int_ = 0.020

#### Refinement

*R*[*F* ^2^ > 2σ(*F* ^2^)] = 0.031
*wR*(*F* ^2^) = 0.078
*S* = 1.04
3975 reflections
156 parameters
2 restraints
H-atom parameters constrained
Δρ_max_ = 0.32 e Å^−3^
Δρ_min_ = −0.20 e Å^−3^
Absolute structure: Flack (1983 ▶), 1919 Friedel pairs
Flack parameter: 0.04 (4)

### 

Data collection: *APEX2* (Bruker, 2009 ▶); cell refinement: *SAINT* (Bruker, 2009 ▶); data reduction: *SAINT*; program(s) used to solve structure: *SHELXTL* (Sheldrick, 2008 ▶); program(s) used to refine structure: *SHELXTL*; molecular graphics: *SHELXTL*; software used to prepare material for publication: *SHELXTL* and *PLATON* (Spek, 2009 ▶).

## Supplementary Material

Crystal structure: contains datablock(s) global, I. DOI: 10.1107/S1600536811026109/lh5274sup1.cif

Structure factors: contains datablock(s) I. DOI: 10.1107/S1600536811026109/lh5274Isup2.hkl

Supplementary material file. DOI: 10.1107/S1600536811026109/lh5274Isup3.cml

Additional supplementary materials:  crystallographic information; 3D view; checkCIF report

## Figures and Tables

**Table 1:** Hydrogen-bond geometry (Å, °) *Cg*1 and *Cg*2 are the centroids of the C1–C6 and C8–C13 benzene rings, respectively.

*D*—H⋯*A*	*D*—H	H⋯*A*	*D*⋯*A*	*D*—H⋯*A*
C12—H12*A*⋯*Cg*1^i^	0.95	2.67	3.3885 (14)	132
C14—H14*A*⋯*Cg*1^ii^	0.98	2.86	3.4853 (14)	124
C2—H2*A*⋯*Cg*2^iii^	0.95	2.73	3.4371 (14)	132
C4—H4*A*⋯*Cg*2^iv^	0.95	2.80	3.5534 (15)	134

Symmetry codes: (i) 
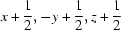
; (ii) 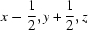; (iii) 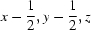; (iv) 
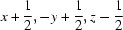
.

**Fig. 1.**
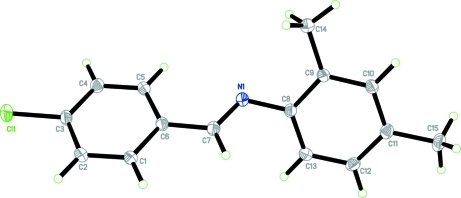
The molecular structure of the title compound showing 50% probability displacement ellipsoids for non-H atoms.

## supplementary crystallographic information

### Comment

The chemistry of the carbon-nitrogen double bond plays a vital role in the progress of science. Schiff-base compounds have been used as fine chemicals and medical substrates. Recently, multi-dentate complexes of iron and nickel showed high activities of ethylene oligomerization and polymerization (Ittel *et al.*, 2000). Schiff bases have a wide variety of applications in many fields, *e.g.*, biological, inorganic and analytical chemistry (Cimerman *et al.*, 2000). They are known to exhibit potent antibacterial, anticonvulsant, anti-inflammatory activities (Shah *et al.*, 1992). In addition, some Schiff bases show pharmacologically useful activities like anticancer (Pandeya *et al.*, 1999), anti-hypertensive and hypnotic (More *et al.*, 2001) properties. Unfortunately, most Schiff bases are chemically unstable and show a tendency to be involved in various equilibria, like tautomeric interconversions, hydrolysis or formation of ionized species (Cimerman & Stefanac, 2001; Galic *et al.*, 1997). Therefore, successful application of Schiff bases requires a careful study of their characteristics.

The molecular structure of the title compound is shown in Fig. 1. The bond lengths (Allen *et al.*, 1987) and angles are within normal ranges. The title compound exists in *trans* configuration with respect to the C7═N1 bond [C7═N1 = 1.2813 (16) Å]. The benzene rings (C1-C6 and C8-C13) form a dihedral angle of 52.91 (6)°.

The crystal structure is stabilized by weak intermolecular C12–H12A···Cg1^i^, C14–H14A···Cg1^ii^, C2–H2A···Cg2^iii^ and C4–H4A···Cg2^iv^ interactions (see Table 1 for symmetry codes), where Cg1 and Cg2 are the centroid of C1-C6 and C8-C13 benzene rings, respectively. No significant classical intermolecular hydrogen bonds are observed.

### Experimental

Equimolar amounts of of 4-chloro benzaldehyde and 2,4 dimethyl aniline were dissolved in a minimum amount of ethanol, followed by addition of 2 ml glacial acetic acid. The solution was refluxed for 8 h then cooled to room temperature and poured into ice cold water. The solid product was collected through filtration and then dried at 353 K. The product was dissolved in ethanol, recrystallized and then dried to give colourless crystals. Yield: 75%, *m.p.* 432-435 K.

### Refinement

All H atoms were positioned geometrically and refined using a riding model with C–H = 0.95 or 0.98 Å and *U*_iso_(H) = 1.2 or 1.5 *U*_eq_(C). A rotating-group model was applied for the methyl group. The highest residual electron density peak is located at 0.69 Å from C12 and the deepest hole is located at 0.15 Å from H15B.

### Crystal data

**Table:**

C_15_H_14_ClN	*F*(000) = 512
*M**_r_* = 243.72	*D*_x_ = 1.269 Mg m^−^^3^
Monoclinic, *C**c*	Mo *K*α radiation, λ = 0.71073 Å
Hall symbol: C -2yc	Cell parameters from 9896 reflections
*a* = 7.2852 (1) Å	θ = 2.7–31.0°
*b* = 15.2715 (2) Å	µ = 0.28 mm^−^^1^
*c* = 11.5382 (1) Å	*T* = 100 K
β = 96.304 (1)°	Block, colourless
*V* = 1275.93 (3) Å^3^	0.40 × 0.24 × 0.20 mm
*Z* = 4	

### Data collection

**Table:**

Bruker SMART APEXII CCD area-detector diffractometer	3975 independent reflections
Radiation source: fine-focus sealed tube	3800 reflections with *I* > 2σ(*I*)
graphite	*R*_int_ = 0.020
φ and ω scans	θ_max_ = 31.1°, θ_min_ = 2.7°
Absorption correction: multi-scan (*SADABS*; Bruker, 2009)	*h* = −10→10
*T*_min_ = 0.898, *T*_max_ = 0.946	*k* = −17→22
14547 measured reflections	*l* = −16→16

### Refinement

**Table:**

Refinement on *F*^2^	Secondary atom site location: difference Fourier map
Least-squares matrix: full	Hydrogen site location: inferred from neighbouring sites
*R*[*F*^2^ > 2σ(*F*^2^)] = 0.031	H-atom parameters constrained
*wR*(*F*^2^) = 0.078	*w* = 1/[σ^2^(*F*_o_^2^) + (0.0435*P*)^2^ + 0.4511*P*] where *P* = (*F*_o_^2^ + 2*F*_c_^2^)/3
*S* = 1.04	(Δ/σ)_max_ = 0.001
3975 reflections	Δρ_max_ = 0.32 e Å^−^^3^
156 parameters	Δρ_min_ = −0.20 e Å^−^^3^
2 restraints	Absolute structure: Flack (1983), 1919 Friedel pairs
Primary atom site location: structure-invariant direct methods	Flack parameter: 0.04 (4)

### Special details

**Table:**

Experimental. The crystal was placed in the cold stream of an Oxford Cryosystems Cobra open-flow nitrogen cryostat (Cosier & Glazer, 1986) operating at 100.0 (1) K.
Geometry. All esds (except the esd in the dihedral angle between two l.s. planes) are estimated using the full covariance matrix. The cell esds are taken into account individually in the estimation of esds in distances, angles and torsion angles; correlations between esds in cell parameters are only used when they are defined by crystal symmetry. An approximate (isotropic) treatment of cell esds is used for estimating esds involving l.s. planes.
Refinement. Refinement of F^2^ against ALL reflections. The weighted R-factor wR and goodness of fit S are based on F^2^, conventional R-factors R are based on F, with F set to zero for negative F^2^. The threshold expression of F^2^ > 2sigma(F^2^) is used only for calculating R-factors(gt) etc. and is not relevant to the choice of reflections for refinement. R-factors based on F^2^ are statistically about twice as large as those based on F, and R- factors based on ALL data will be even larger.

### Fractional atomic coordinates and isotropic or equivalent isotropic displacement parameters (Å^2^)

**Table:**

	*x*	*y*	*z*	*U*_iso_*/*U*_eq_	
Cl1	1.00011 (4)	0.620805 (19)	0.24699 (3)	0.02374 (8)	
N1	0.98639 (15)	0.21574 (7)	0.00510 (9)	0.0164 (2)	
C1	1.10819 (17)	0.36449 (8)	0.25068 (11)	0.0164 (2)	
H1A	1.1660	0.3226	0.3036	0.020*	
C2	1.09740 (18)	0.45134 (9)	0.28532 (11)	0.0174 (2)	
H2A	1.1469	0.4693	0.3612	0.021*	
C3	1.01230 (18)	0.51134 (8)	0.20614 (11)	0.0172 (2)	
C4	0.93749 (17)	0.48677 (9)	0.09481 (11)	0.0180 (2)	
H4A	0.8786	0.5288	0.0425	0.022*	
C5	0.95032 (17)	0.39987 (8)	0.06141 (11)	0.0166 (2)	
H5A	0.9011	0.3823	−0.0147	0.020*	
C6	1.03527 (17)	0.33777 (8)	0.13907 (11)	0.0152 (2)	
C7	1.04654 (18)	0.24507 (8)	0.10622 (11)	0.0163 (2)	
H7A	1.1011	0.2049	0.1628	0.020*	
C8	0.99097 (17)	0.12365 (8)	−0.01051 (11)	0.0153 (2)	
C9	1.05963 (17)	0.08957 (8)	−0.11026 (11)	0.0151 (2)	
C10	1.05936 (17)	−0.00135 (8)	−0.12576 (11)	0.0164 (2)	
H10A	1.1084	−0.0250	−0.1920	0.020*	
C11	0.98889 (18)	−0.05863 (8)	−0.04642 (11)	0.0177 (2)	
C12	0.91927 (18)	−0.02310 (9)	0.05076 (11)	0.0182 (2)	
H12A	0.8702	−0.0608	0.1052	0.022*	
C13	0.92063 (18)	0.06723 (8)	0.06920 (11)	0.0172 (2)	
H13A	0.8735	0.0905	0.1363	0.021*	
C14	1.1312 (2)	0.14980 (9)	−0.19821 (12)	0.0212 (3)	
H14A	1.1739	0.1151	−0.2615	0.032*	
H14B	1.2342	0.1844	−0.1603	0.032*	
H14C	1.0321	0.1892	−0.2301	0.032*	
C15	0.9863 (2)	−0.15650 (9)	−0.06663 (13)	0.0244 (3)	
H15A	0.8998	−0.1840	−0.0183	0.037*	
H15B	1.1104	−0.1803	−0.0457	0.037*	
H15C	0.9469	−0.1686	−0.1490	0.037*	

### Atomic displacement parameters (Å^2^)

**Table:**

	*U*^11^	*U*^22^	*U*^33^	*U*^12^	*U*^13^	*U*^23^
Cl1	0.03157 (16)	0.01487 (12)	0.02478 (15)	0.00105 (13)	0.00319 (11)	−0.00480 (12)
N1	0.0188 (5)	0.0143 (5)	0.0163 (5)	−0.0001 (4)	0.0033 (4)	−0.0014 (4)
C1	0.0181 (6)	0.0165 (5)	0.0144 (5)	−0.0008 (4)	0.0007 (4)	−0.0001 (4)
C2	0.0194 (6)	0.0181 (5)	0.0144 (5)	−0.0012 (5)	0.0011 (4)	−0.0029 (4)
C3	0.0189 (6)	0.0133 (5)	0.0198 (6)	−0.0009 (4)	0.0045 (5)	−0.0034 (4)
C4	0.0188 (6)	0.0163 (6)	0.0187 (6)	0.0012 (4)	0.0010 (4)	0.0005 (4)
C5	0.0187 (6)	0.0161 (5)	0.0149 (5)	0.0001 (4)	0.0015 (4)	−0.0014 (4)
C6	0.0159 (5)	0.0141 (5)	0.0157 (5)	−0.0004 (4)	0.0025 (4)	−0.0017 (4)
C7	0.0168 (5)	0.0145 (5)	0.0178 (5)	0.0002 (4)	0.0024 (4)	−0.0004 (4)
C8	0.0162 (5)	0.0134 (5)	0.0160 (6)	−0.0001 (4)	0.0009 (4)	−0.0009 (4)
C9	0.0164 (5)	0.0149 (5)	0.0138 (5)	−0.0010 (4)	0.0011 (4)	−0.0012 (4)
C10	0.0178 (5)	0.0160 (5)	0.0155 (5)	0.0003 (4)	0.0016 (4)	−0.0025 (4)
C11	0.0185 (6)	0.0146 (5)	0.0191 (6)	−0.0012 (5)	−0.0019 (5)	−0.0005 (4)
C12	0.0198 (6)	0.0168 (6)	0.0180 (6)	−0.0029 (5)	0.0011 (5)	0.0026 (4)
C13	0.0204 (6)	0.0173 (6)	0.0141 (5)	−0.0008 (5)	0.0024 (4)	−0.0005 (4)
C14	0.0272 (7)	0.0173 (6)	0.0205 (6)	−0.0027 (5)	0.0088 (5)	−0.0008 (5)
C15	0.0305 (7)	0.0135 (6)	0.0284 (7)	−0.0016 (5)	−0.0005 (6)	−0.0020 (5)

### Geometric parameters (Å, °)

**Table:**

Cl1—C3	1.7418 (13)	C8—C9	1.4043 (17)
N1—C7	1.2813 (16)	C9—C10	1.3999 (17)
N1—C8	1.4187 (15)	C9—C14	1.5045 (18)
C1—C2	1.3900 (17)	C10—C11	1.4036 (18)
C1—C6	1.3988 (17)	C10—H10A	0.9500
C1—H1A	0.9500	C11—C12	1.3906 (19)
C2—C3	1.3908 (18)	C11—C15	1.5125 (18)
C2—H2A	0.9500	C12—C13	1.3957 (18)
C3—C4	1.3906 (17)	C12—H12A	0.9500
C4—C5	1.3881 (18)	C13—H13A	0.9500
C4—H4A	0.9500	C14—H14A	0.9800
C5—C6	1.4012 (17)	C14—H14B	0.9800
C5—H5A	0.9500	C14—H14C	0.9800
C6—C7	1.4702 (17)	C15—H15A	0.9800
C7—H7A	0.9500	C15—H15B	0.9800
C8—C13	1.3980 (18)	C15—H15C	0.9800
			
C7—N1—C8	116.85 (11)	C10—C9—C14	121.06 (11)
C2—C1—C6	121.02 (12)	C8—C9—C14	120.48 (11)
C2—C1—H1A	119.5	C9—C10—C11	122.03 (12)
C6—C1—H1A	119.5	C9—C10—H10A	119.0
C1—C2—C3	118.36 (11)	C11—C10—H10A	119.0
C1—C2—H2A	120.8	C12—C11—C10	118.34 (12)
C3—C2—H2A	120.8	C12—C11—C15	120.68 (12)
C4—C3—C2	122.02 (12)	C10—C11—C15	120.98 (12)
C4—C3—Cl1	118.84 (10)	C11—C12—C13	120.76 (12)
C2—C3—Cl1	119.13 (10)	C11—C12—H12A	119.6
C5—C4—C3	118.88 (12)	C13—C12—H12A	119.6
C5—C4—H4A	120.6	C12—C13—C8	120.39 (12)
C3—C4—H4A	120.6	C12—C13—H13A	119.8
C4—C5—C6	120.52 (11)	C8—C13—H13A	119.8
C4—C5—H5A	119.7	C9—C14—H14A	109.5
C6—C5—H5A	119.7	C9—C14—H14B	109.5
C1—C6—C5	119.20 (11)	H14A—C14—H14B	109.5
C1—C6—C7	119.47 (11)	C9—C14—H14C	109.5
C5—C6—C7	121.33 (11)	H14A—C14—H14C	109.5
N1—C7—C6	123.24 (12)	H14B—C14—H14C	109.5
N1—C7—H7A	118.4	C11—C15—H15A	109.5
C6—C7—H7A	118.4	C11—C15—H15B	109.5
C13—C8—C9	120.00 (11)	H15A—C15—H15B	109.5
C13—C8—N1	120.80 (12)	C11—C15—H15C	109.5
C9—C8—N1	119.14 (11)	H15A—C15—H15C	109.5
C10—C9—C8	118.47 (11)	H15B—C15—H15C	109.5
			
C6—C1—C2—C3	−0.07 (19)	C7—N1—C8—C9	−133.76 (13)
C1—C2—C3—C4	0.46 (19)	C13—C8—C9—C10	−1.42 (18)
C1—C2—C3—Cl1	−179.11 (10)	N1—C8—C9—C10	−178.57 (11)
C2—C3—C4—C5	−0.81 (19)	C13—C8—C9—C14	178.43 (12)
Cl1—C3—C4—C5	178.77 (10)	N1—C8—C9—C14	1.29 (18)
C3—C4—C5—C6	0.76 (19)	C8—C9—C10—C11	1.54 (18)
C2—C1—C6—C5	0.04 (19)	C14—C9—C10—C11	−178.31 (12)
C2—C1—C6—C7	−178.99 (12)	C9—C10—C11—C12	−0.63 (19)
C4—C5—C6—C1	−0.39 (19)	C9—C10—C11—C15	178.60 (11)
C4—C5—C6—C7	178.62 (12)	C10—C11—C12—C13	−0.40 (18)
C8—N1—C7—C6	−174.26 (12)	C15—C11—C12—C13	−179.64 (13)
C1—C6—C7—N1	−178.57 (12)	C11—C12—C13—C8	0.50 (19)
C5—C6—C7—N1	2.4 (2)	C9—C8—C13—C12	0.44 (19)
C7—N1—C8—C13	49.12 (17)	N1—C8—C13—C12	177.53 (12)

### Hydrogen-bond geometry (Å, °)

**Table:**

Cg1 and Cg2 are the centroids of the C1–C6 and C8–C13 benzene rings, respectively.

**Table:**

*D*—H···*A*	*D*—H	H···*A*	*D*···*A*	*D*—H···*A*
C12—H12A···Cg1^i^	0.95	2.67	3.3885 (14)	132
C14—H14A···Cg1^ii^	0.98	2.86	3.4853 (14)	124
C2—H2A···Cg2^iii^	0.95	2.73	3.4371 (14)	132
C4—H4A···Cg2^iv^	0.95	2.80	3.5534 (15)	134

Symmetry codes: (i) *x*+1/2, −*y*+1/2, *z*+1/2; (ii) *x*−1/2, *y*+1/2, *z*; (iii) *x*−1/2, *y*−1/2, *z*; (iv) *x*+1/2, −*y*+1/2, *z*−1/2.
